# Editorial: Ferroptosis and regulation mechanism in the tumor immune microenvironment for tumor progression and treatment

**DOI:** 10.3389/fimmu.2025.1748341

**Published:** 2025-12-09

**Authors:** Yuliang Wei, Jinghua Pan, Lu Wang, Yunlong Pan

**Affiliations:** 1 Department of General Surgery, The First Affiliated Hospital of Jinan University, Guangzhou, Guangdong, China; 2 Hepatobiliary surgery Department, The Fifth Affiliated Hospital of Jinan University (Shenhe People’s Hospital), Heyuan, Guangdong, China; 3 State Key Laboratory of Bioactive Molecules and Druggability Assessment, and Minister of Education Key Laboratory of Tumor Molecular Biology, Institute of Precision Cancer Medicine and Pathology, School of Medicine, Jinan University, Guangzhou, Guangdong, China

**Keywords:** ferroptosis, tumor immune microenvironment, biomarker, tumor, treatment

The field of ferroptosis research has burgeoned over the past decade, revealing profound implications for cancer biology and therapy. This Research Topic, *“Ferroptosis and regulation mechanism in the tumor immune microenvironment for tumor progression and treatment*,” compiles pioneering work elucidating the multifaceted roles of ferroptosis across diverse malignancies. This topic that has rapidly evolved to reveal critical insights for cancer progression and therapy. This Research Topic underscores how ferroptosis—an iron-dependent form of cell death driven by lipid peroxidation—serves as a pivotal regulator in oncology, bridging molecular mechanisms with clinical applications. Collectively, these studies demonstrate that targeting ferroptosis can disrupt cancer resilience, yet they also emphasize the complexity of its interactions with immune cells and therapeutic agents, setting the stage for transformative advances in precision medicine.

## Ferroptosis: a molecular crossroads in cancer

Ferroptosis—an iron-dependent cell death driven by lipid peroxidation—intersects critical pathways in tumorigenesis, immunity, and therapy resistance. Wang et al. demonstrate that *FASN* shields colorectal cancer stem cells (CSCs) from ferroptosis by inhibiting SREBP2 activation, thereby sustaining stemness and tumor growth. Similarly, Ge et al. summarized ferroptosis to breast cancer chemoresistance, highlighting the GSH-GPX4 and FSP1-CoQ10 axes as targets to overcome anti-apoptotic defenses. These studies underscore that cancer cells co-opt ferroptosis-suppressive mechanisms to evade death, positioning ferroptosis induction as a therapeutic imperative.

## Immune microenvironment: ferroptosis as a double-edged sword

Ferroptosis dynamically interacts with the tumor immune microenvironment (TIME). While triggering ferroptosis can amplify anti-tumor immunity, it also risks inflammatory collateral damage. Shu et al. reveal that *ARIH2* in hepatocellular carcinoma (HCC) correlates with immune infiltration, ferroptosis sensitivity, and immune checkpoint expression, suggesting its dual role as a biomarker and immunomodulator. Conversely, gastric cancer studies caution that ferroptosis inducers Erastin may paradoxically fuel metastasis via oxidative stress (Zhao et al.). This duality necessitates precision strategies to identify non-coding RNAs as regulators of ferroptosis-dependent lipid peroxidation in liver cancer, offering tissue-specific targets to mitigate off-tumor toxicity (1). Therefore, Ferroptosis is a double-edged sword for anti-cancer and need to identify precision biomarkers to application its scenario, especially in TIME.

## Ferroptosis in diagnostics and therapeutic synergy

Several articles spotlight ferroptosis-related biomarkers for early detection and prognosis. Wang et al. leverage multi-omics to develop a 7-gene HCC diagnostic model (centered on *TRIB3* and *NQO1*) that predicts immunotherapy response. In leukemia, Zhong et al. identify a 5-FRG signature (*ACSL6*, *SLC11A2*, etc.) diagnosing chronic myeloid leukemia CMLCML and sensitizing drug-resistant cells to imatinib via ferroptosis induction. These findings illustrate how ferroptosis pathways refine risk stratification and expand therapeutic options.

Notably, ferroptosis inducers synergize powerfully with existing modalities. Thyroid cancer (Liu et al.) and NSCLC (Zhang et al.) studies emphasize combining ferroptosis inducers RSL3 with immune checkpoint blockade, leveraging TIME remodeling to enhance cytotoxicity. Zhang et al. further note that nanomedicine and AI-driven drug delivery could optimize this synergy, heralding a new era of combinatorial precision therapy.

Despite these promising developments, significant challenges remain, including the cell-type specificity of ferroptosis mechanisms, which vary across cancer lineages and immune contexts, complicating broad application. Biomarker validation is another hurdle, as multi-omics signatures require rigorous prospective clinical testing to ensure reliability. Safety concerns also persist, particularly regarding off-target ferroptosis in normal tissues, which could limit therapeutic utility and necessitate innovative delivery systems for tumor-selective induction.

Future research must prioritize mechanistic depth to elucidate spatiotemporal crosstalk between ferroptosis, inflammation, and immune checkpoints, while advancing personalized approaches through integrated biomarker profiling. Innovations in nanomedicine and AI-driven delivery hold promise for optimizing combination therapies, ultimately transforming ferroptosis modulation into a cornerstone of oncology. This Research Topic collectively affirms that harnessing ferroptosis within the tumor immune microenvironment offers a paradigm for improving cancer treatment, with the potential to redefine therapeutic landscapes through continued interdisciplinary collaboration.
